# Whole-body diffusion magnetic resonance imaging with simultaneous multi-slice excitation in children and adolescents

**DOI:** 10.1007/s00247-023-05622-9

**Published:** 2023-03-15

**Authors:** Paul-Christian Krueger, Martin Krämer, Thomas Benkert, Sophia Ertel, Ulf Teichgräber, Matthias Waginger, Hans-Joachim Mentzel, Katja Glutig

**Affiliations:** 1 Section Paediatric Radiology, Department of Radiology, Jena University Hospital, Am Klinikum 1, Jena, Germany; 2 Department of Radiology, Jena University Hospital, Jena, Germany; 3 MR Application Predevelopment, Siemens Healthcare GmbH, Erlangen, Germany; 4 Clinic for Radiology – Focus Pediatric Radiology, University of Münster and University Hospital Münster, Albert-Schweitzer-Campus 1 – Building A1, Münster, Germany

**Keywords:** Adolescent, Child, Diffusion magnetic resonance imaging, Retrospective studies, Signal-to-noise ratio, Whole-body imaging

## Abstract

**Background:**

Whole-body magnetic resonance imaging (WB-MRI) is an increasingly used guideline-based imaging modality for oncological and non-oncological pathologies during childhood and adolescence. While diffusion-weighted imaging (DWI), a part of WB-MRI, enhances image interpretation and improves sensitivity, it also requires the longest acquisition time during a typical WB-MRI scan protocol. Interleaved short tau inversion recovery (STIR) DWI with simultaneous multi-slice (SMS) acquisition is an effective way to speed up examinations.

**Objective:**

In this study of children and adolescents, we compared the acquisition time, image quality, signal-to-noise ratio (SNR) and apparent diffusion coefficient (ADC) values of an interleaved STIR SMS-DWI sequence with a standard non-accelerated DWI sequence for WB-MRI.

**Materials and methods:**

Twenty children and adolescents (mean age: 13.9 years) who received two WB-MRI scans at a maximum interval of 18 months, consisting of either standard DWI or SMS-DWI MRI, respectively, were included. For quantitative evaluation, the signal-to-noise ratio (SNR) was determined for b800 images and ADC maps of seven anatomical regions. Image quality evaluation was independently performed by two experienced paediatric radiologists using a 5-point Likert scale. The measurement time per slice stack, pause between measurements including shim and total measurement time of DWI for standard DWI and SMS-DWI were extracted directly from the scan data.

**Results:**

When including the shim duration, the acquisition time for SMS-DWI was 43% faster than for standard DWI. Qualitatively, the scores of SMS-DWI were higher in six locations in the b800 images and four locations in the ADC maps. There was substantial agreement between both readers, with a Cohen’s kappa of 0.75. Quantitatively, the SNR in the b800 images and the ADC maps did not differ significantly from one another.

**Conclusion:**

Whole body-MRI with SMS-DWI provided equivalent image quality and reduced the acquisition time almost by half compared to the standard WB-DWI protocol.

## Introduction

Whole-body magnetic resonance imaging (WB-MRI) is an imaging technique for visualization of the entire body and is used primarily in childhood and adolescence to avoid other imaging modalities that use ionizing radiation [1]. The national German S1 guideline “Whole Body Magnetic Resonance Imaging in Childhood and Adolescence,” which was most recently modified in 2021, highlights the diagnostic value of WB-MRI in this age range [2]. The Taskforce Oncology Guidelines of the European Society for Paediatric Radiology confirm their significance on a global scale [3]. WB-MRI is highly valuable in the diagnosis of oncological conditions such as Hodgkin’s and non-Hodgkin’s lymphomas [4], the diagnosis of distant disease in osteo-, Ewing and soft tissue sarcomas [5] and in Langerhans cell histiocytosis [6] and for the monitoring of tumour predisposition syndromes [7]. Additionally, WB-MRI is performed for radiation-free diagnosis in infancy and adolescence of non-oncological disorders such as chronic non-bacterial osteomyelitis and chronic recurrent multifocal osteomyelitis [8, 9]. Other important indications include fever of unknown origin [10] and complications of sickle cell disease [11].

Currently, there is no standard protocol for whole-body imaging [12]. However, WB-MRI with diffusion-weighted imaging (DWI) has been shown to increase diagnostic accuracy in cases of residual lymphoma in adults [13]. Diffustion-weighted imaging in WB-MRI can provide additional information on diffusivity, highlighting changes in the cell density of different tissues, in addition to features seen on standard sequences such as short tau inversion recovery (STIR) and T1 contrast. [14]. This can be helpful in oncological diagnostics for initial staging, treatment response and follow-up monitoring [15]. A particular problem with diffusion-weighted sequences in WB-MRI is the long acquisition time. Younger children have difficulty lying motionless for long periods and older (larger) children may require more slice stacks to achieve whole-body coverage. Diffusion-weighted imaging alone can require more than 20 min to cover the entire body depending on the size of the patient and the chosen slice thickness. Diffusion-weighted imaging with a quicker acquisition time would, therefore, be a clear benefit in paediatric WB-MRI.

One possibility of sequence acceleration is the simultaneous excitation and readout of multiple slices (SMS) by exploiting the different spatial sensitivity profiles of multi-coil arrays [16, 17]. This technique has already been established for MRI [18], and its advantages have been outlined in various studies on different organ systems, such as the liver [19], breast [20] and skeletal system [21], but mainly in adult patients. Kenkel et al. showed both a drastic reduction in the duration of whole-body MRI using SMS-DWI and its clinical applicability in adults [22]. Tabari et al. and, more recently, Glutig et al. demonstrated the benefit of simultaneous multi-slice abdominal MRI during childhood and adolescence using the kidney as an example in patients with cystic fibrosis and tuberous sclerosis complex [23, 24]. The use of an efficient interleaving scheme for WB STIR DWI imaging [2, 25] represents another method of reducing the acquisition time of WB-MRI.

This study aimed to investigate the feasibility and clinical utility of an interleaved WB-STIR DWI-SMS sequence in childhood and adolescence.

## Materials and methods

### Study design

All children with a clinical indication for WB-MRI who underwent at least two whole-body examinations between January 2020 and July 2021 were included in the study. The indications for recurrent WB-MRI included oncologic and non-oncologic follow-up and screening for tumour predisposition syndromes. The interval between the current WB-MRI and the earlier examination was 18 months or less. The follow-up whole-body examination used a vendor-supplied “works in progress” (WIP) simultaneous multi-slice diffusion-weighted sequence (SMS-DWI). For reasons of compliance, only one WB-DWI investigation was performed on a single date. This study was approved by the local ethics committee (Reg. No. 2022–2600). All patients or their legal guardians provided written informed consent for the examination.

### Patient measurements

All MRI examinations were performed on a clinical 1.5-tesla (T) whole body MRI system (MAGNETOM Sola, Siemens Healthineers, Erlangen, Germany) using the following vendor-supplied clinical coils for WB-MRI: 20-channel cranial-neck coil, 12-channel BioMatrix body coil, 18-channel body coil, 36-channel peripheral angio coil, and 32-channel spine coil. The patients underwent a WB-MRI examination consisting of a coronal short tau inversion recovery turbo spin echo (STIR TSE), a coronal T_1_ SPACE (sampling perfection with application optimized contrast using different flip angle evolution) and an axial whole-body diffusion-weighted sequence. During the first WB-MRI, a standard DWI (sDWI) was used; in the follow-up scan, DWI was performed using the prototype SMS technique with enabled interleaved STIR module (SMS-DWI). All DWI sequences used an echo-planar imaging (EPI) readout. The acquisition parameters for both sequences are listed in Table 1. Except for repetition duration (TR), which could be shortened by the SMS approach, the parameters of the diffusion-weighted sequences were the same. All sequences were acquired while breathing freely. Depending on patient size, only the number of slice stacks of DWI was adjusted as necessary, while keeping the number of slice stacks constant between the two scans performed for each patient.

**Table 1:** Summary of sequence parameters

Sequence parameters	sDWI	SMS DWI
Breathing scheme	free-breathing	free-breathing
Slice thickness/gap (mm)	6/0	6/0
Slices	50	50
Repetition time (TR in ms)	7190	3470
Echo time (TE in ms)	63	63
Bandwidth (Hz/pixel)	1925	1925
Field of view (FOV) read (mm^2^)	500	500
FOV phase (%)	80	80
GRAPPA acceleration factor	2	2
SMS acceleration factor	-	2
Matrix	268 × 216	268 × 216
Voxel size	1.9 × 1.9	1.9 × 1.9
Diffusion preparation *b*-values (averages):	50 (4), 800 (12), calculated 1400	
Flip angle (°)	90	90

Summary of sequence parameters for standard diffusion weighted imaging (sDWI) and new simultaneous multi-slice (SMS) DWI for one stack each. Number of stacks varied depending on height of the patient. *DWI* diffusion-weighted imaging, *GRAPPA* generalized autocalibrating partial parallel acquisition

### Assessment of acquisition time

To compare the acquisition times of sDWI and SMS-DWI, the digital imaging and communications in medicine (DICOM) tags of all the acquired imaging series were analyzed retrospectively. For this purpose, the start time and sequence duration of each layer stack were extracted, whereas the start time of the subsequent layer stack was subtracted from those of its predecessor. From this information, the total time for the acquisition of a one-layer stack, including all scan adjustments and shimming, as well as the duration of only the sDWI and SMS-DWI sequences, were extracted.

### Assessment of image quality

Qualitative evaluation was performed using Mint-Lesion (MINT Medical GmbH, Heidelberg, Germany) by two paediatric radiologists, one with 10 years (P.C.K.) and the other with 15 years (K.G.) of paediatric imaging experience. Using a 5-point Likert scale (1 = very good, 2 = good, 3 = adequate, 4 = poor, 5 = insufficient), the image quality assessment of b800 and the ADC parameter maps of the sDWI and SMS-DWI were performed independently. The readers were blinded to the patient data and sequences used.

**Fig. 1:**
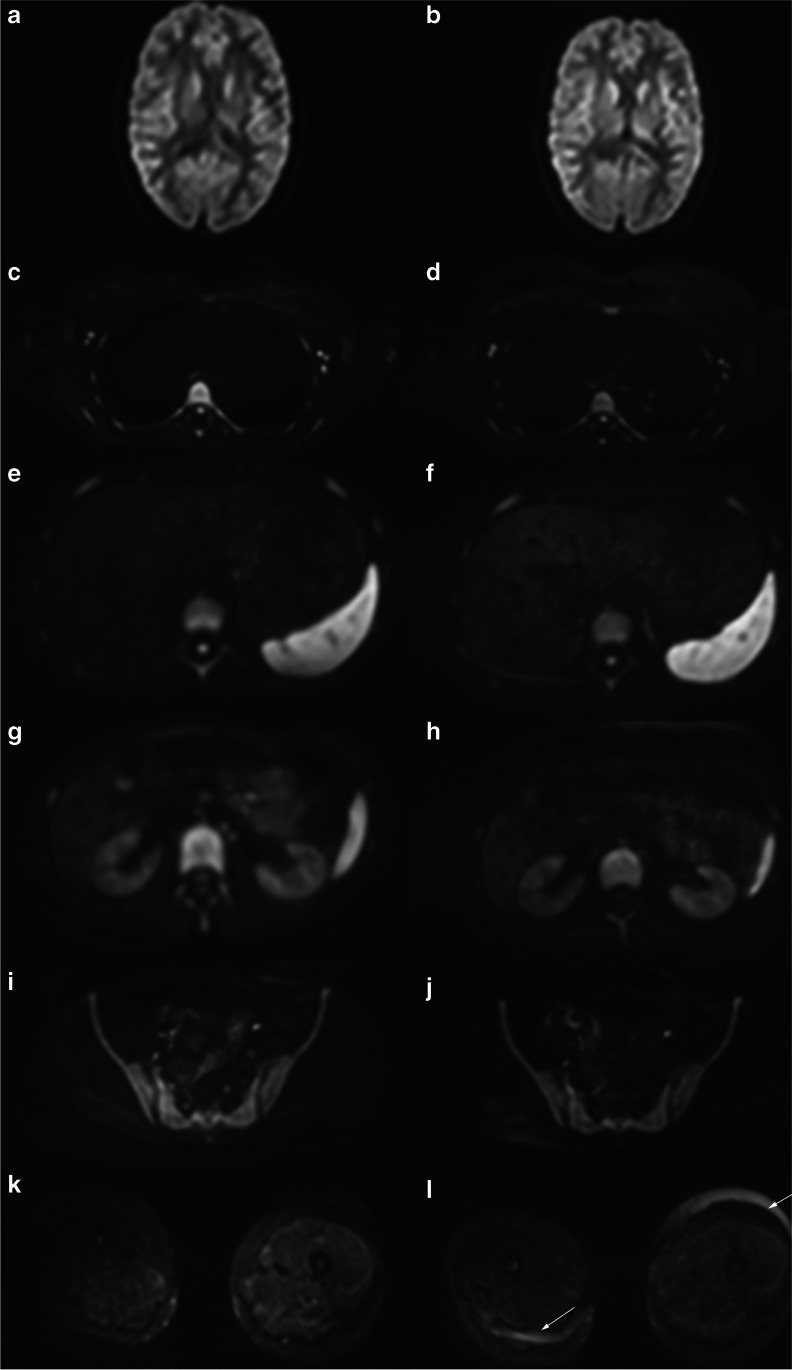
Whole-body-magnetic resonance scan in a 16-year-old girl with fever of unknown origin. Comparison of axial orientated diffusion-weighted examinations at b800 for brain (**a**,**b**), chest (**c**,**d**), upper abdomen (**e**,**f**), kidneys (**g**,**h**), pelvis (**i**,**j**) and thigh muscles (**k**,**l**). **a**,**c**,**e**,**g**,**i**,**k** Standard diffusion-weighted imaging (DWI). **b**,**d**,**f**,**h**,**j**,**l** Simultaneous multi-slice DWI. Note small field inhomogeneities of the subcutaneous fat of the limb, probably due to limited compatibility of the used coils with the simultaneous multi-slice technique (arrows in **l**)

**Fig. 2:**
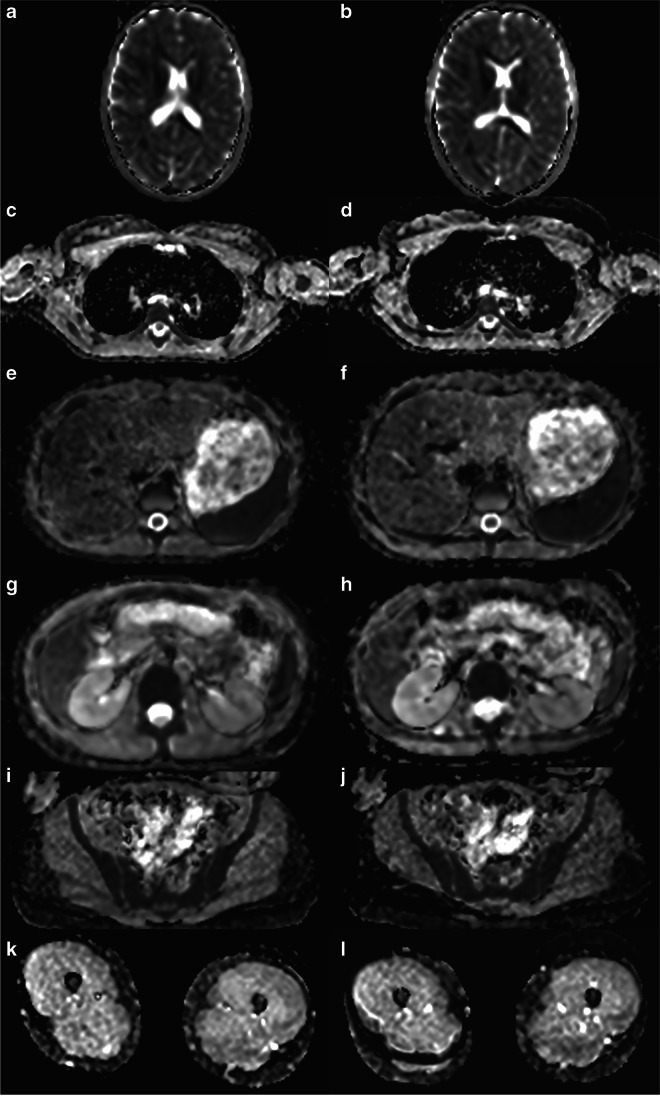
Comparison of axial orientated ADC (apparent diffusion coefficient) parameter map for brain (**a**,**b**), chest (**c**,**d**), upper abdomen (**e**,**f**), kidneys (**g**,**h**), pelvis (**i**,**j**) and thigh muscles (**k**,**l**) for the same 16-year-old girl shown in Fig. 1 with fever of unknown origin. **a**,**c**,**e**,**g**,**i**,**k** Standard diffusion-weighted imaging (DWI), **b**,**d**,**f**,**h**,**j**,**l** Simultaneous multi-slice-DWI. Overall comparable image quality

Image quality was assessed for each of the following locationst: brain, chest, trunk and extremities for each patient (Figs. 1 and 2). Particular attention to distortion-free imaging and artifacts was noted as well as general image quality.

### Quantitative assessment of signal-to-noise ratio and apparent diffusion coefficient

For quantitative evaluation, regions of interest (ROI) were placed in seven different areas, both at b = 800 s/mm^2^ and on the ADC parameter map. The areas were chosen according to the respective regions and included the white matter, cerebrospinal fluid (CSF), mediastinum, liver, spleen, kidneys and thigh muscles. The sizes of the ROI were adjusted for the target localization. The Mint-Lesion software ensured the same size and location of ROI for different b800 and ADC images. For each ROI, the mean value, standard deviation (SD) and maximum value were automatically acquired via the Mint Lesion, and the SNR was calculated for b800.

### Statistical analysis

Statistical analysis was performed using Python programming language (Python Software Foundation, https://python.org/) and statsmodels [26, 27].

Ratings, ADC and SNR were recorded as mean and SD. Descriptive statistics were used to summarize the population characteristics and image findings. Scores for the image quality of sDWI and SMS-DWI were compared using the Wilcoxon signed-rank test. Interrater agreement was determined using Cohen’s kappa (correlation: < 0.2 poor; 0.2–0.4 fair; 0.4–0.6 moderate; 0.6–0.8 substantial; > 0.8 almost perfect).

## Results

### Clinical characteristics

A total of twenty children and adolescents (9 male) were included in the main study. The mean age was 13.9 years ± 5.4 years (range 5–18 years). Table 2 shows the demographic data of the patients divided into different diagnostic groups.

**Table 2:** Clinical characteristics of recruited patients (*n*=20) divided into different diagnostic groups

Diagnosis	Age (year) + SD	Height (cm) + SD	Number *n* (%)
AML/ALL	11.3 ± 3.8	1.34 ± 0.2	4 (20)
Cancer predisposition	12.7 ± 3.1	1.40 ± 0.1	4 (20)
Solid tumours	11.0 ± 4.9	1.38 ± 0.3	3 (15)
Lymphoma	17.0 ± 0.8	1.70 ± 0.1	3 (15)
FUO	15.2 ± 2.6	1.55 ± 0.1	2 (10)
CRMO	14.0	1.56	1 (5)
Langerhans cell histiocytosis	13.5 ± 1.5	1.62 ± 0.1	2 (10)
Sickle cell anaemia	8.0	1.30	1 (5)

*AML* acute myeloid leukaemia, *ALL* acute lymphatic leukaemia, *CRMO* chronic recurrent multifocal osteomyelitis, *FUO* fever of unknown origin, *SD* standard deviation

### Measuring times

The sDWI took an average of 3 min 56 s ± 12 s per stack, of which 1 min 31 s ± 12 s was used for the shim and 2 min 25 s was used for acquisition. The average time for SMS-DWI was 2 min 15 s ± 6 s for each stack, which was broken down into 1 min 28 s for acquisition and 47 s ± 6 s for the shim. As a result, the overall measurement time for a patient undergoing the acquisition of six-layer stacks was 13 min 29 s ± 0:35 min for SMS-DWI and 23 min 38s ± 01:15 min for sDWI.

Using the SMS-DWI resulted in a 43% reduction in examination time and a reduction of 39% in sequence acquisition time. Table 3 provides an overview of the examination times for patients depending on the two different diffusion-weighted sequences used for WB-MRI.

**Table 3:** Patient measurements—comparison of examination times between sDWI and SMS-DWI, acquisition time, additional time for shimming (shim) and total time (total) for 6 stacks, all in minutes and seconds with standard deviation (SD)

Sequence	Acquisition time	Shim + SD	Total + SD (min)
sDWI	14 min 30 s	09 min 08 s ± 75 s	23 min 38 s ± 75 s
SMS-DWI	08 min 48 s	04 min 36 s ± 35 s	13 min 29 s ± 35 s

*min* minutes, *SD* standard deviation, *sDWI* standard diffusion-weighted imaging, *SMS* simultaneous multi-slice

### Qualitative analysis–subjective image quality

#### Interobserver agreement–interrater variability–Cohen’s kappa

On average the Cohen’s kappa of both readers was substantial (0.75). There was substantial agreement with respect to the image quality (IQ) of the DWI b800 images and almost perfect agreement with respect to the ADC. Further details are provided in Table 4.

**Table 4:** Interobserver variability between rater 1 and rater 2 for qualitative analysis of sDWI and SMS-DWI

Categories	Cohen’s kappa	95% CI
IQ b800		
sDWI	0.68	0.59–0.76
SMS-DWI	0.79	0.72–0.86
IQ ADC		
sDWI	0.83	0.77–0.90
SMS-DWI	0.89	0.84–0.95

*ADC* apparent diffusion coefficient; *CI* confidence intervals; Cohen´s Kappa (correlation: < 0.2 poor; 0.2–0.4 fair; 0.4–0.6 moderate; 0.6–0.8 substantial; > 0.8 almost perfect); *IQ* image quality; *sDWI* standard diffusion weighted image; *SMS-DWI* simultaneous multi-slice DWI

#### Mean ratings

Both sequences showed subjectively good and comparable image quality for diffusion weightings as well as for the ADC parameter maps. The mean ratings of the sDWI were slightly lower than those of the SMS-DWI. Overall, the SMS-DWI was preferred in six out of seven ratings for the b800 image and in four out of seven ratings for the ADC, with significantly better ratings for the white matter in the brain (b800 and ADC), liver (b800), CSF (ADC) and kidney (b800 and ADC). Significantly lower ratings of SMS-DWI were observed only for the limb muscles in the b800 images. Table 5 provides an overview of the scores for the DWI with b800 and Table 6 shows the scores for the ADC parameter values.

**Table 5:** Average ratings of the DWI-weighted images (b800), Wilcoxon signed rank test, *P* < 0.05 significant

Region	Reader 1			Reader 2		
	sDWI	SMS-DWI	*P*-value	sDWI	SMS-DWI	*P*-value
CSF	2.1 ± 0.2	2.1 ± 0.4	0.705	2.1 ± 0.2	2.0 ± 0.4	0.705
WM	2.8 ± 0.5	2.5 ± 0.6	0.035 ^a^	2.7 ± 0.5	2.4 ± 0.7	0.035^a^
Mediastinum	2.8 ± 0.5	2.5 ± 0.6	1.000	2.7 ± 0.5	2.4 ± 0.6	0.083
Liver	2.4 ± 0.6	2.1 ± 0.2	0.020 ^a^	2.4 ± 0.5	2.1 ± 0.2	0.008 ^a^
Spleen	1.5 ± 0.6	1.2 ± 0.4	0.096	1.4 ± 0.5	1.3 ± 0.4	0.083
Kidney	1.4 ± 0.5	1.0 ± 0.0	0.008 ^a^	1.3 ± 0.5	1.1 ± 0.2	0.025 ^a^
Thigh muscles	2.0 ± 0.0	2.1 ± 0.0	0.059	2.0 ± 0.0	2.1 ± 0.0	0.059

Data is presented as mean values with standard deviation. ^a^ indicates the significant values

*CSF* cerebrospinal fluid;* sDWI* standard diffusion weighted image

*SMS-DWI* simultaneous multi-slice DWI; *WM* white matter

**Table 6:** Average ratings of the ADC maps, Wilcoxon signed rank test, *P* < 0.05 significant

Region	Reader 1			Reader 2		
	sDWI	SMS-DWI	*P*-value	sDWI	SMS-DWI	*P*-value
CSF	2.6 ± 0.6	2.0 ± 0.3	< 0.001 ^a^	2.6 ± 0.6	2.0 ± 0.2	0.001^a^
WM	4.0 ± 0.2	3.2 ± 0.4	< 0.001 ^a^	3.9 ± 0.4	3.2 ± 0.4	< 0.001 ^a^
Mediastinum	3.0 ± 0.2	3.1 ± 0.2	0.157	2.9 ± 0.3	3.1 ± 0.2	0.083
Liver	2.9 ± 0.3	2.8 ± 0.4	0.317	3.0 ± 0.5	3.0 ± 0.5	1.000
Spleen	2.1 ± 0.3	2.2 ± 0.5	0.705	2.1 ± 0.3	2.2 ± 0.5	0.705
Kidney	1.7 ± 0.5	1.1 ± 0.3	0.001 ^a^	1.9 ± 0.5	1.2 ± 0.4	0.002 ^a^
Thigh muscles	2.0 ± 0.0	2.1 ± 0.2	0.165	2.0 ± 0.0	2.1 ± 0.2	0.317

Data is presented as mean values with standard deviation. ^a^ indicates the significant values

*ADC* apparent diffusion coefficient; *CSF* cerebrospinal fluid; *sDWI* standard diffusion weighted image; *SMS-DWI* simultaneous multi-slice DWI; *WM* white matter

**Fig. 3:**
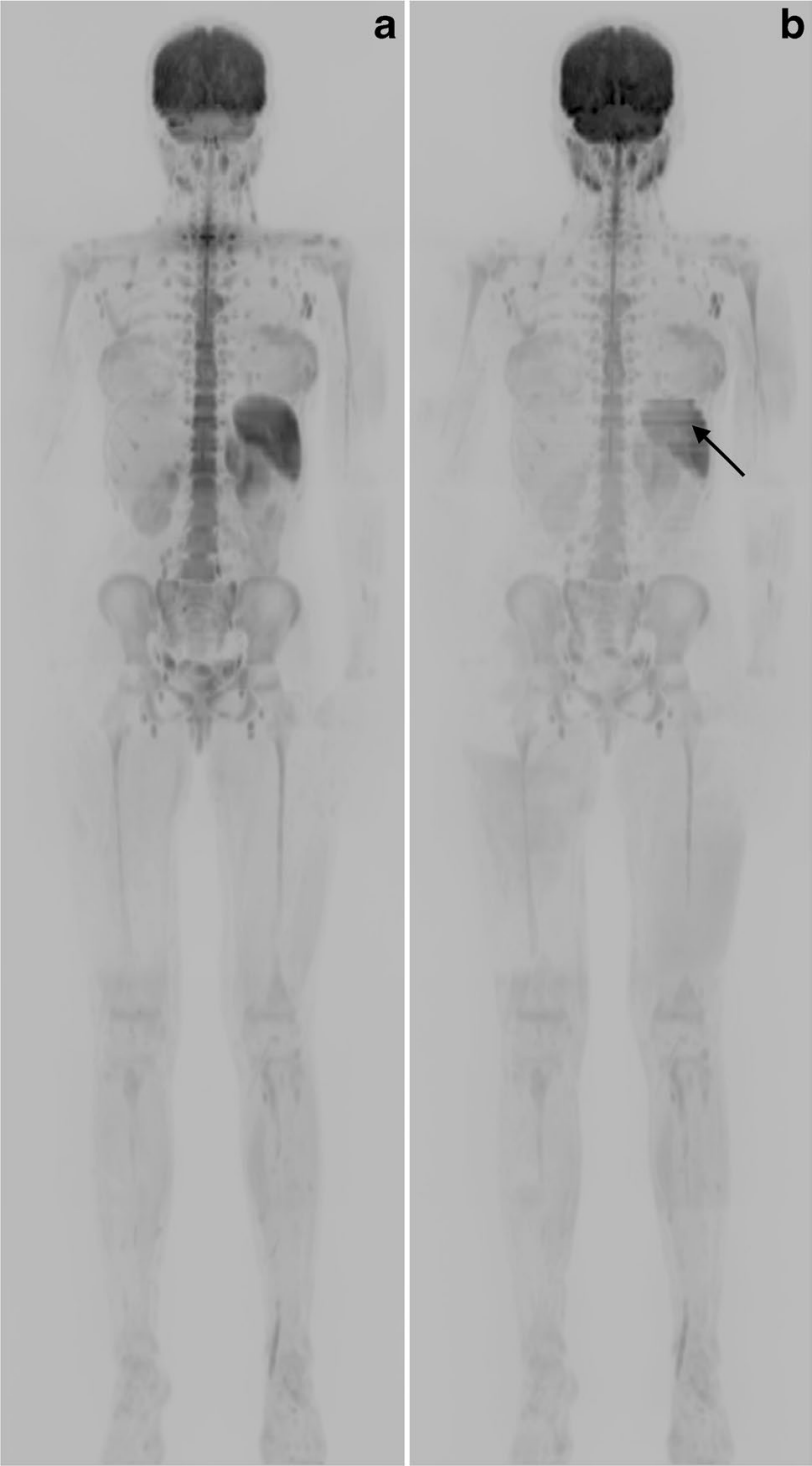
Comparison of the coronal 3-dimensional reconstructions for standard diffusion-weighted imaging (sDWI) (**a**) and simultaneous multi-slice diffusion-weighted imaging (SMS-DWI) (**b**) in a 15-year-old girl with cancer preposition syndrome. Both measurements with 6 stacks of DWI inline composed on the scanner. There are visible step formations in the SMS-DWI due to the different shimming algorithm without slice adjustments (black arrow). Overall, however, there is comparable quality with a homogeneous signal

For a better overview, the combined DWI stacks were reconstructed as a composite 3-dimensional (D) dataset and displayed using an inverted grayscale colormap. Qualitative evaluation showed that the SMS-DWI data exhibited noticeable step artifacts in the reconstruction along the slice direction. Figure 3 shows a comparison of the 3-D composite reconstruction of a colormap inverted b800 image from a 15-year-old girl with tumour predisposition syndrome.

### Quantitative analysis

**Fig. 4:**
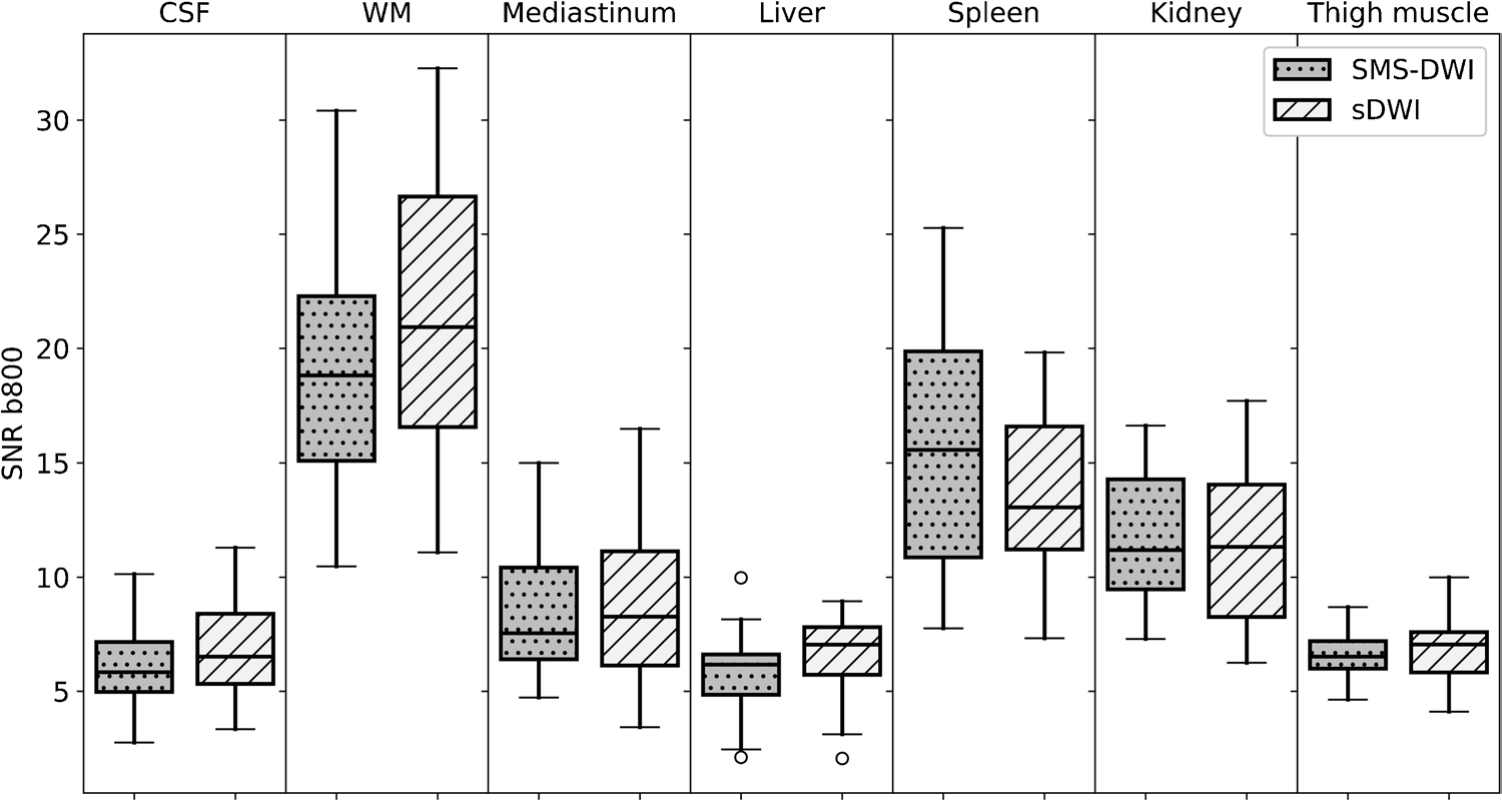
Boxplot of signal to noise ratio (SNR) values measured in b800 in 20 patients comparing sDWI und SMS-DWI. There is no significant difference in the measured b800 values in the regions studied. *CSF* cerebrospinal fluid; *sDWI* standard diffusion weighted image; *SMS-DWI *simultaneous multi-slice DWI; *WM* white matter

The SNR values of sDWI and SMS-DWI at b800 showed no significant differences for all the assessed regions (white matter, CSF, chest, liver, kidney, and thigh muscle). Figure 4 demonstrates the SNR values for b800 for the assessed regions as a boxplot.

**Fig. 5:**
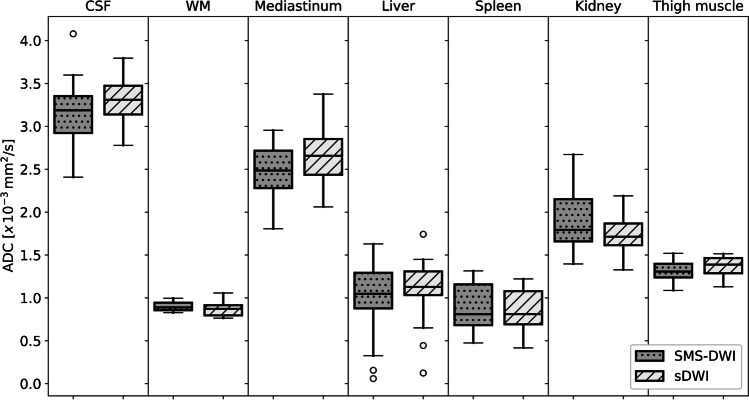
Boxplot of ADC values measured in 20 patients comparing sDWI und SMS-DWI. There is no significant difference in the measured ADC values in the regions studied. *ADC* apparent diffusion coefficient; *CSF* cerebrospinal fluid; *sDWI* standard diffusion weighted image; *SMS-DWI* simultaneous multi-slice DWI; *WM* white matter

There were no significant differences in the recorded ADC in the investigated regions, except for the right kidney, which showed significantly higher values on the SMS-DWI. Figure 5 presents the ADC of the assessed regions, comparing sDWI and SMS-DWI.

An overview of the measured ADC for sDWI and SMS-DWI and a comparison with published values is given in Table 7.

**Table 7:** ADC values as mean with SD for sDWI and SMS-DWI compared to the literature

Organ	sDWI ADC	SMS-DWI ADC	Literature reference
CSF	3.29 ± 0.28	3.13 ± 0.41	3.06 ± 0.19 [34]
WM	0.84 ± 0.14	0.86 ± 0.08	0.76 ± 0.05 [35]
Liver	1.09 ± 0.35	0.99 ± 0.44	1.28 ± 0.12 [36]
Spleen	0.85 ± 0.22	0.87 ± 0.26	0.81 ± 0.13 [36]
Kidney	1.72 ± 0.22	1.91 ± 0.35	2.26 ± 0.37 [37]
Thigh muscles	1.36 ± 0.11	1.31 ± 0.12	1.77 ± 0.36 [﻿^34^]

*ADC* apparent diffusion coefficient, *CSF* cerebrospinal fluid, *SD* standard deviation, *sDWI* standard diffusion weighted image, *SMS-DWI* simultaneous multi-slice DWI, *WM* white matter

## Discussion

Analysis of the results demonstrates that for WB-MRI, the STIR-SMS diffusion sequence (SMS-DWI) reduces the examination time by an average of 43% compared with our previously used standard diffusion sequence without SMS acceleration. A faster examination time was achieved by combining a modern interleaved scheme in the SMS technique with an SMS factor of 2, in addition to parallel imaging with a GRAPPA acceleration factor of 2, which was used by both sequences. Image quality, SNR and ADC values were comparable to those of standard DWI.

The acceleration of the examination time achieved was remarkable compared to other studies in adults. Kenkel et al. showed a 24.2–25.9% reduction in examination time using a slice-acceleration factor of 3 with the same image quality and ADC values [22]. Currently, we are not aware of any work that has systematically investigated the duration of WB-DWI measurements during childhood and adolescence.

The SMS technique allows the simultaneous excitation of slices and thus a significant acceleration in data acquisition time depending on the number of excited slices [16]. Compared to conventional parallel imaging, there is only a slight potential SNR penalty and minor effects on TE when using SMS. Setsompop et al. demonstrated the application of the SMS technique as one of the first for diffusion-weighted imaging techniques [28]. Taron et al. investigated the SMS-DWI for whole-body positron-emission tomography/MRI with a reduction in scan time of 40% [29] and similarly for abdominal MRI [19, 30]. They were able to show a substantial reduction in liver DWI scan time (70%), with comparable image quality. Recently, regarding abdominal MRI in children and adolescents with cystic fibrosis, Glutig et al. showed a 32% reduction in examination time with no decrease in SNR using SMS-DWI and improved image quality by additional reconstruction with motion-correction [24]. In this work, the combination of STIR-DWI and the SMS technique for whole-body imaging was used for the first time.

In addition to the intrinsic sequence acquisition time, other factors such as the number of layer stacks and the time spent shimming before each layer stack must be considered when analyzing the total examination time of the DWI sequence. In this study, the total measurement time was significantly reduced by decreasing the sequence-only measurement time by 39% and the shim time between layer stacks by 46%. The shorter shim time results from the fact that the SMS-DWI sequence is based on a simple volume shim and is not compatible with the whole-body shim “SliceAdjust” [31] of the MRI device manufacturer, as is the case with the standard DWI sequence. As a result of the more simplified shim algorithm, typical inhomogeneities and image artefacts, such as “broken spine artefacts”, may occur more frequently [31].

The degree of interobserver agreement in image quality evaluations showed that the ratings in our study were quite trustworthy. Our quantitative analysis did not reveal any appreciable variations in the SNR of b800 images. In this study, the ADC values showed no significant differences in six of seven localizations analyzed. This is in agreement with the results of Glutig et al. [24] and Xu et al. [32, 33], but contrasts with the data of Taron et al. [19], who measured lower ADC values.

This study has some limitations. First, only a small number of patients were identified. Second, it was not a homogeneous group of patients with the same disease, and we did not examine focal or specific pathologies, but only compared the examination time, general image quality, SNR and ADC values. Furthermore, this was a retrospective study from a single centre, which may have introduced some bias. All patients included in the study had already received a WB-MRI examination with the standard DWI. Because of the standardization of the examinations, we could use these data for the study and no patient had to receive an MRI examination solely for the study. The maximum interval between the current SMS-DWI and the sDWI was 18 months; a lot can happen in those 18 months, especially in oncology patients.

Large tumours or lymphadenopathy on initial or follow-up MRI may affect image quality. However, this was not the case in the included patients; disease-related findings were only detected in one examination.

This study was performed as part of routine clinical surveillance imaging. In future research, we aim to take practical steps, such as applying artificial intelligence and deep learning, to address deficiencies and to broaden the results of this study.

## Conclusion

Whole body-MRI with interleaved STIR SMS-DWI provided equivalent image quality and significantly reduced acquisition time, which is important in paediatric patients and should therefore replace the previous sDWI sequence.

## Data Availability

The datasets generated during and/or analyzed during the current study are available from the corresponding author on reasonable request.
